# Improving medical students' competencies: using longitudinal ambulatory setting preceptorship

**DOI:** 10.15694/mep.2019.000015.1

**Published:** 2019-01-21

**Authors:** Javad Kojuri, Mohammad Hossein Rahmani, Reza Golchin Vafa, Amin Ahmadi, Mohammad Javad Mehdizadeh, Reza Heidar Alizadeh, Bardia Zamiri, Hossein Fatemian, Morteza Akbari, Sajad Kargar, Solmaz Zare, Leila Bazrafcan, Mitra Amini

**Affiliations:** 1 Clinical Education Research Center

**Keywords:** ambulatory clinic, preceptorship, medical students

## Abstract

This article was migrated. The article was marked as recommended.

## Letter

A preceptorship is an optional experience in which an experienced physician gives a personal guide and supervision to medical students during the first or second year of medical school. Preceptorships offer the preclinical medical student good opportunity to track a patient over time and to experience a real clinical setting. The results of previous articles about preceptorship showed that the students report that this method can be an effective way of preparing them for clinical training and motivation for studying basic sciences. Besides it provides a unique opportunity to see the patients in follow up visits. ( Tan 2011, Willoughby 2016).

The present experience was carried out in Shiraz medical school that is one of the oldest and best ranked medical school in south Iran ( Nasr 2009). In this experience, a combination of shadowing program for first year medical students, Individual preceptorship (IP) stage 1 for second and third year medical students and IP stage 2 for clinical students was done. All of the preceptorship supervision was done by an experience full professor cardiologist who was also an expert in the field of medical education. A well-educated general practitioner was also selected as a perceptor. A total number of 8 first years and 8 second and third years and 10 medical students in clinical setting participated voluntarily in this program.

For first year medical students shadowing program was designed by observing doctor-patient communication. This shadowing experience offers the first year medical student a chance for seeing their future career, knowing the realities of medicine, and realizing the importance of professionalism.

IP during stage 1 pairs each second or third year medical student with the general practitioner to work in an ambulatory patient care setting every week for four hours.

Students communicate with patients to get medical histories, perform correct physical examinations and advice patients under the supervision of the general practitioner. Students learn through observation and supervision from this general practitioner, who provides them feedback to help them develop the capabilities in effective communication with patients. Each patients encounter was recorded after written permission from the patients, so students are able to see the recorded encounters and understand their mistakes.

Another purpose for these sessions for medical students is to motivate them for learning basic science. For example, as students are learning cardiovascular physiology, they get a detailed history from a real patient with cardiac disease and they examine the heart in the clinical setting. An interactive e- learning material about electrocardiogram was developed by experienced cardiologists to help them understand the normal and abnormal cardiac rhythms. They also learn about the social determinant of health in the real setting.

IP during stage 2 pairs students in the clinical setting with an experience full professor clinical faculty. In this stage, the students obtain the history, perform the physical examination, suggest the differential diagnosis and write prescription under the direct supervision of the experienced faculty. This ambulatory setting has a large electronic database of patients, so there is an opportunity for these students to do research based on electronic patients registry by ethically approved proposals. The experienced faculty also teaches students about illness scripts to improve their critical thinking and clinical reasoning ability. He divides teaching sessions into short segments to help teaching and learning fit a busy schedule. He Reviews each patient para clinic test results and treatment plans with the student, and then have the student give the follow-up and educational instructions to the patients. The students were asked to write their reflections after visiting a patient and review important learning points ( Audétat 2017).

There is a good opportunity for all medical students in this ambulatory clinic to longitudinally observe the patients and learn about the continuity of care.

The results of the present study showed some important points. The students reported that this opportunity prepared them for improving clinical skills. They also believed that this program helped them to correlate the basic sciences concepts with patient care. Students reported a sensation that they were a respected part of the health care team and required the capability to reflect on their own achievements. Students reported six areas of this program that they thought were beneficial: the experience of different types of clinical practices, differential diagnosis, patient electronic records, professionalism, role modeling and visiting varieties of patients. Preceptors ascertained the value of educating students in the early years of medical school. They pronounced acquisition of self-appreciation for their teaching and were invigorated by the achievement of the students. These two preceptors showed interest in to recruit other preceptors among their colleagues.

In conclusion, our preliminary results showed that focused preceptorship in the ambulatory setting with frequent observations of patients as well as supervision with preceptor physicians will help our future doctors in their future career. Larger samples and future data collection is needed to measure these students’ success in doing their future responsibilities as a physician.

## Notes On Contributors

**Dr. Javad Kojuri** is a full Professor of Cardiology and Clinical Education Research Center. His interest is in the field of cardiology and medical education. (ORCHID: https://orcid.org/0000-0001-8909-897X).

**Dr. Mitra Amini** is a full Professor of Community Medicine and Clinical Education Research Center in Shiraz University of Medical Sciences. Her interest is in the field of medical education and medical education research.(ORCHID: https://orcid.org/0000-0002-7332-5151).

**Dr. Solmaz Zare** and **Dr. Leila Bazrafcan** have a Ph.D. in medical education. Other authors are medical students except for **Mr. Morteza Akbari** and **Mr. Sajad Kargar** who are engineers.
