# Supplementary material for: Improving medical students' competencies: using longitudinal ambulatory setting preceptorship
Source: MedEdPublish (2016). 2019 Jan 21;8:15. [Version 1] doi: 10.15694/mep.2019.000015.1 (PMC10712517; doi:10.15694/mep.2019.000015.1)
Supplement: Peer review history

# Reviewer report: Reviewer response for version 1

**DOI:** 10.21956/mep.19750.r29627

**Published:** 2019-06-23

**Authors:** Ken Masters

**Affiliations:** 1 Sultan Qaboos University

This review has been migrated. The reviewer awarded 4 stars out of 5 A short and interesting letter on improving medical students' competencies using a longitudinal ambulatory setting preceptorship. The letter gives details of how the preceptorship functions across the medical degree, and is specifically tailored to the medical students’ current levels. One can surmise (and, indeed, it appears to be supported by the results) that such an exercise will allow students to more easily relate their current work to medical practice, and to gain deeper insight into what will be expected from them as qualified health professionals.One point of criticism: there are many, albeit minor, language errors, and these frequently interfere with the communication; it would have been a good idea if the authors had more carefully proofed their letter to avoid these errors.In spite of this, an interesting letter, and I look forward to seeing a more detailed and fully-fledged research article on the topic.

Reviewer Expertise:

NA

No decision status is available

# Reviewer report: Reviewer response for version 1

**DOI:** 10.21956/mep.19750.r29626

**Published:** 2019-01-25

**Authors:** Trevor Gibbs

**Affiliations:** 1 AMEE

This review has been migrated. The reviewer awarded 4 stars out of 5 This paper describes what many might consider to be a standard approach to a curriculum; an interactive programme that combines structured learning within a community setting, over a period time during undergraduate medical education. Although not wishing to degrade either the school or its faculty, nor its international setting, I feel that this is a major development in its geographical setting and was probably not an easy activity to create, with many obstacles.I would wish to congratulate them on this initiative and would hope that the authors would now provide a more descriptive paper which explores the obstacles to and the solutions the authors provided to develop such an important programme.

Reviewer Expertise:

NA

No decision status is available
